# Sovereign default network and currency risk premia

**DOI:** 10.1186/s40854-023-00485-3

**Published:** 2023-05-04

**Authors:** Lu Yang, Lei Yang, Xue Cui

**Affiliations:** 1 College of Economics, Shenzhen University, 3688 Nanhai Avenue, Nanshan District, Shenzhen, 518060 Guangdong People’s Republic of China; 2 Shanghai Jiaotong University, Shanghai Advanced Institute of Finance, 211 West Huaihai Road, Shanghai, 200030 People’s Republic of China

**Keywords:** Sovereign CDS, Currency risk premia, High-dimensional network, LASSO, G01, G12, G15

## Abstract

We construct a sovereign default network by employing high-dimensional vector autoregressions obtained by analyzing connectedness in sovereign credit default swap markets. We develop four measures of centrality, namely, degree, betweenness, closeness, and eigenvector centralities, to detect whether network properties drive the currency risk premia. We observe that closeness and betweenness centralities can negatively drive currency excess returns but do not exhibit a relationship with forward spread. Thus, our developed network centralities are independent of an unconditional carry trade risk factor. Based on our findings, we develop a trading strategy by taking a long position on peripheral countries’ currencies and a short position on core countries’ currencies. The aforementioned strategy generates a higher Sharpe ratio than the currency momentum strategy. Our proposed strategy is robust to foreign exchange regimes and the coronavirus disease 2019 pandemic.

## Introduction

The modern financial system was developed based on the fiat money guaranteed by governments or creditable authorities (i.e., the Federal Reserve). As the cornerstone of the financial system, fiat money plays a crucial role in the globalization of goods and capital. However, measuring its real value is difficult because sovereign credit is invisible. Consequently, the foreign exchange market compares the value of national currencies to the US dollar, which is determined by the trading of market participants. As a simple, unconditional arbitrage strategy, the use of the carry trade has been thoroughly documented by examining the uncovered interest rate parity (UIP). However, less is known about its sources of risk when the carry trade is still profitable. The sovereign credit swap (CDS) market enables the direct assessment of government credit, thereby allowing us to evaluate its relationship with currency risk premia.

Predicting currency returns is difficult. Various strategies, such as carry and momentum, have been developed to predict these returns by considering the cross-sectional and time-series predictability of currency returns (Menkhoff et al. 2012). Unlike a carry trader, a momentum trader rarely pays attention to macroeconomic fundamental signals without incorporating any systematic risk. However, as currency is created, it comoves with the macroeconomic fluctuations that occur over time. Currency returns are predicted using numerous macroeconomic indicators, including the global political risk factor (Filippou et al. 2018), economic activity factor (Dahlquist and Hasseltoft 2020), and sovereign risk factor (Augustin et al. 2020; Chernov et al. 2021). The finance literature proposed a wide variety of factors, which prompted efforts to identify the key driver of currency risk premia.

Mathematically, the combination of the aforementioned factors results in the relative importance of one country in the global economy or network centrality as per network theory. Theoretically, Richmond (2019) documented that countries’ trade network centrality is the fundamental driver of currency risk premia in terms of carry trade returns and currency excess returns. During a recession, core countries exhibit low currency risk premia. However, in the Twin Ds (default and devaluation) framework (Na et al. 2018), the effects of a default on exchange rates are unclear. Augustin et al. (2020) demonstrated that default asymmetry affects currency devaluation between core and periphery countries. The covariance risk of default (contagion) dominated during the 2012 European debt crisis. Considering that the probability of default is low, estimating the covariance risk of sovereign default is difficult. We developed network centrality measures to determine the effects of covariance risk on currency risk premia from sovereign default networks.

We first consider that the default risk of a government, indicated by its sovereign CDS, can be transmitted to other countries, to bridge the sovereign default network and currency risk premia (Chen et al. 2020; Chang et al. 2021; Corte, et al. 2021). For example, the European debt crisis was triggered by the default of the Greek government, which then rapidly spread to other European countries. Therefore, the sovereign risk spillover among countries allows us to construct a sovereign default network to explore its role in currency risk premia. Moreover, although interest rate parity provides a straightforward method to calculate the forward exchange rate, the compositions of interest rate differentials have not been explored. Credit differentials of currencies should be a primary research focus. Furthermore, the availability of international capital for one country strongly influences its interest rate. Combining all the gaps in the related literature, we investigate the effect of network centrality, which is defined as the role of one country in the sovereign default network, on currency risk premia. The reason is that this effect reflects not only the credit differential but also the connections with the international financial market.

We utilize the forecast error variance decomposition under a high-dimensional vector autoregression (VAR) model incorporating a Least Absolute Shrinkage and Selection Operator (LASSO) to estimate connectedness in the sovereign CDS market. This method is an effective way for evaluating the risk spillover in the sovereign CDS market (Alessi and Savona 2021; Balduzzi et al. 2022; Bostanci and Yilmaz 2020; Diebold and Yilmaz 2014). Then, we develop a sovereign default network and calculate the dynamic changes of network centrality. According to network theory, we develop four network centrality measures, namely, degree, closeness, betweenness, and eigenvector centralities, to indicate the source of currency risk premia.

The main findings reveal that closeness and betweenness centralities significantly explain currency excess returns but not the forward spread. Based on these findings, we develop a decile portfolio by sorting the degree of closeness centrality. The long–short strategy involves taking long positions in the currency of peripheral countries and short positions in the currencies of core countries. This strategy provides an annualized return of 211 basis points with a Sharpe ratio of 0.172. Considering that the network centrality factor (NCF) is independent of the carry trade risk, we employ the momentum risk factor (MOM) to construct the momentum portfolio as comparison groups instead. This process generates an annualized return of 1.914% with a Sharpe ratio of 0.126. Moreover, we identify that NCF and MOM exhibit similar explanations on the currency excess return across the deciles.

The NCF can be considered the transformer between a country-specific (idiosyncratic) risk and a global (systematic) risk to gain intuition (Acemoglu et al. 2015). In a well-diversified currency portfolio, the foreign country-specific risk averages out, and the domestic investor holding this portfolio is compensated only for bearing its own country-specific and global risks. However, in the Twin Ds framework, a country-specific risk may transform into a global risk owing to the spillover effect or financial contagion. In contrast to the carry trade returns that rely on the average growth rate of aggregate consumption across countries (Lustig et al. 2011), our proposed trading strategy based on the NCF is strongly correlated with the changes in global financial market volatility, particularly for the extreme events (Fan et al. 2022). Indeed, the trading strategy based on the MOM provides high returns under the volatile market condition, which is the main reason to employ the MOM as the benchmark.

This study makes three contributions to the relevant literature. First, we map the global sovereign default network through the connectedness measurement while considering the shock induced by the coronavirus disease 2019 (COVID-19) pandemic by employing the high-dimensional VAR model. Unlike the structure of the international trade network, our findings suggest that the structure of the sovereign default network changes dynamically, particularly under the shock induced by the COVID-19 pandemic. Second, we develop network centrality measures to account for the covariance risk of default and explore its role in the Twin Ds. Our findings entail implications regarding network structures because they relate to the sovereign credit risk spillover mechanisms that activate when currency risk premia are high. Given the rarity of default events, our analysis provides an alternative view of the Twin Ds from a network perspective. Third, we provide the first empirical evidence that the network centrality of the sovereign default network significantly influences currency excess returns but not the forward spread. In other words, our proposed NCF is independent of the carry trade but is associated with momentum trading. Our proposed trading strategy based on the NCF is more profitable than the momentum strategy, which proves that the NCF can explain the MOM. This result also indicates that the network centrality of the sovereign default network is a true driver of momentum trading.

The remainder of this paper is arranged as follows. Section “Literature review” provides a brief literature review. Section “Data and methodology” presents the data and methodology. Section “Empirical results” discusses the empirical findings and related applications. Section “Conclusion” concludes this study.

## Literature review

Sovereign risk is intrinsically related to exchange rate fluctuation. A depreciation of a currency always implies a decrease in confidence in the credit of the issuing country (Doshi et al. 2017; Na et al. 2018). The triggering of a sovereign CDS contract and the collapse of the currency value are rare events. Hence, the relevant literature focused on the risk spillover between the sovereign CDS and the foreign exchange market, which has a strong time-varying pattern (Hui and Fong 2015; Augustin et al. 2020; Feng et al. 2021). Augustin et al. (2020) revealed that substantial common movement occurs in the global sovereign CDS market although the cross-sectional variation mainly originates from the government debt. By contrast, our proposed network centrality is a direct measure of the importance of one country in the sovereign default network, which can be considered the source or bridge of risk spillover. Thus, the proposed network centrality provides a broad view of the relationship between sovereign risk and currency risk premia.

Allen and Gale’s (2000) pioneering research on the financial network suggested that no idiosyncratic risk exists within the complete network because idiosyncratic risk instantaneously transforms into systematic risk. Moreover, Allen and Gale (2007) observed that the network structure changes when a financial crisis occurs. Acemoglu et al. (2015) also provided similar evidence by suggesting that densely connected networks are more resilient to sudden markets shock than sparsely connected ones. In particular, the sudden COVID-19 pandemic outbreak has had different effects on the sovereign CDS market and foreign exchange market. The pandemic has dramatically changed the network structure of the sovereign default network and its effect on the currency excess return. Therefore, our study also provides straightforward evidence of how the structure of the sovereign default network changes under different market conditions.

One should first test whether the UIP holds globally to measure currency risk premia (Fama 1984) because the interest rate is a useful indicator of the economic conditions and sovereign risk of a country. Next, specific indicators for the relevant country need to be developed to describe its currency returns. Hassan (2013) provided evidence that the size of the economy is an important pricing factor of cross-sectional currency returns. In addition, a larger economy always pays lower expected returns. Furthermore, uncertainty about future mean interest rates is the reason for cross-sectional violations of the UIP and the key driver for carry trades (Hassan and Mano 2019; Husted et al., 2017). Ismailov and Rossi (2018) empirically demonstrated that when uncertainty is low, the UIP still holds most of the time. Nonetheless, uncertainty can be observed in many forms, such as macroeconomic uncertainty (Berg and Mark 2018; Yang et al. 2018), commodity price uncertainty (Ready et al. 2017; Yang et al. 2017; Yang 2022), and sovereign risk (Augustin et al. 2020; Bernoth and Herwartz 2021; Corte et al. 2021; Galice and Zeng 2021).

The aforementioned studies failed to assess directly the network centrality of the sovereign default network for explaining currency excess returns although the NCF is not a pricing factor for carry trade returns. As suggested by Yamani (2019), currency momentum can serve as a diversification role in carry trades. Therefore, we refer to the literature on currency momentum because our proposed network centrality measures are similar to the comovement of assets. Menkhoff et al. (2012) proposed the currency momentum strategy, which generates an annualized return of 10% by taking a long–short position on the past winner and loser currencies. The currencies in the winner and loser groups exhibit a strong comovement pattern under a short-term period. Menkhoff et al. (2012) noted that a higher country risk usually brings higher positive excess returns to its currency. Similarly, the proposed NCF can also significantly explain the MOM. Furthermore, Daniel and Moskowitz’s (2016) proposal may not function as the market declines because its volatility is high. Dahlquist and Hasseltoft (2020) also demonstrated that the return of currency momentum was low following the 2008 global financial crisis. The aforementioned findings drive us to explore the effect of the COVID-19 pandemic on currency excess returns. The outcomes determined for the COVID-19 pandemic are opposite to those for the normal period.

Finally, we find that the existence of currency excess returns is driven by idiosyncratic risk by combining studies on the sovereign default network and currency risk premia. Chen et al. (2020) also documented similar findings in their study of the sovereign CDS market. They provided direct evidence that the idiosyncratic contagion occurs in the European sovereign CDS market through the conditional VAR approach. By contrast, according to the connectedness measure proposed by Diebold and Yilmaz (2014), Bostanci and Yilmaz (2020) concluded that capital flow is the key factor in determining the network structure of the global sovereign CDS market. The aforementioned studies focused exclusively on the sovereign CDS market. Thus, our investigation provides the first empirical evidence to explain dynamically the influence of network centrality in the sovereign default network on currency excess returns.

## Data and methodology

### Data sources and sample

We consider all the 5 year sovereign CDSs actively traded in the market from January 2, 2011, to December 31, 2020, according to the daily frequency obtained from Thomson Reuters’ Datastream, to construct a sovereign default network. The currency is US dollars.^1^ We take the first difference of log sovereign CDS prices to calculate the returns. Then, we construct a sovereign default network based on the daily log returns. In particular, given that the euro was adopted in 1999, we only consider the DataStream Europe 5-year sovereign CDS index to denote the area covered by the euro. Therefore, our dataset includes 41 countries because of the availability of spot (forward) foreign exchange data. Table 1 specifies the currencies of these countries.

**Table 1:** Descriptions of samples

Country	Location	Regime	Abbr.	Country	Location	Regime	Abbr.
ARGENTINE	South America	FTT	ARS	MALAYSIA	Asia	FTT	MYR
AUSTRALIA	Oceania	FFT	AUD	MEXICO	North America	FFT	MXN
BAHRAIN	Asia	FFT	BHD	MOROCCO	Africa	CPG	MAD
BRAZIL	South America	FTT	BRL	NEW ZEALAND	Oceania	FTT	NZD
BULGARIA	Europe	CON	BGN	NORWAY	Europe	FFT	NOK
CANADA	North America	FFT	CAD	PERU	South America	FTT	PEN
CHILE	South America	FFT	CLP	PHILIPINES	Asia	FTT	PHP
CHINA	Asia	FTT	CNH	POLAND	Europe	FFT	PLN
COLOMBIA	South America	FTT	COP	QATAR	Asia	CPG	QAR
CROATIA	Europe	STR	HRK	ROMANIA	Europe	FTT	RON
CZECH	Europe	FTT	CZK	RUSSIA	Europe	FFT	RUB
DENMARK	Europe	CPG	DKK	SAUDI ARABIA	Asia	CPG	SAR
Euro Area	Europe	FFT	EUR	SERBIA	Europe	CLA	RSD
Hong Kong	Asia	FFT	NOR	SOUTH AFRICA	Africa	FTT	ZAR
HUNGARY	Europe	FTT	HUF	SWEDEN	Europe	FFT	SEK
ICELAND	Europe	FTT	ISK	THAILAND	Asia	FTT	THB
INDONESIA	Asia	STR	IDR	TUNISIE	Africa	CLA	TND
ISRAEL	Asia	FTT	ILS	TURKEY	Europe	FTT	TRY
JAPAN	Asia	FFT	MEX	UK	Europe	FFT	GBP
KAZAKHSTAN	Asia	FTT	KZT	VIETNAM	Asia	STR	VND
KOREA	Asia	FTT	KRW				

The foreign exchange regimes are classified according to IMF Annual Report on Exchange Arrangements and Exchange Restrictions 2019. FFT denotes freely floating, FTT denotes floating, CON denotes currency board, STR denotes stabilized arrangement, CPG denotes conventional peg, and CLA denotes crawl-like arrangement. Meanwhile, CON, STR, CPG, and CLA are treated as exchange rate anchor framework

Following previous research (Richmond 2019), the monthly currencies’ excess returns and forward spreads are calculated according to end-of-month foreign exchange rates. Similar to sovereign CDS prices, all foreign exchange rates are denoted by US dollars. For the control variables, we follow the approach of Augustin et al. (2020), who considered that government and total external debts jointly influence the sovereign default network and sovereign exchange rate. The data adopted in the present study are mainly collected from the Oxford Economics Global Data Workstation. In some cases, government short-term external debt is collected from central bank statistics. The research sample includes all countries for which the quarterly sovereign CDS market, monthly foreign exchange, and quarterly external debt data are available. Consequently, we interpolate all the quarterly data into monthly values to match the monthly currency risk premia.^2^ The final dataset contains 4920 monthly observations across 41 countries.

**Table 2:** Variable definitions

Variable name	Description
Dependent variable	
Currency excess return	The one-month currency excess returns computed by the approach in section “Emphirical model”
Currency forward spread	The log difference between forward exchange rate and spot exchange rate based on the US dollar
Network centrality	
Degree centrality	A measure of the weighted sum of all edges given a node. Similarly, in-strength measures the weighted sum of all edges from other nodes to the given node, while out-strength measures the weighted sum of all edges from the given node to other nodes
Closeness centrality	The degree of closeness centrality is calculated by taking the sum of reciprocals of the length of the shortest path between the relevant node and all others
Betweenness centrality	A measure of all the shortest paths in the network through a given node in the fraction form. A large number of shortest paths indicates that the relevant node has a high value of betweenness centrality
Eigenvector centrality	The importance of a node in a network by considering the centrality of its connected nodes. A high eigenvector centrality node is observed when it connects to other high eigenvector centrality nodes
Sovereign characteristics	
Government debt ratio	(Government external debt)/(Total external debt)
Total debt ratio	(Total external debt)/GDP

### Measuring network centrality

We use the approaches of Demirer et al. (2017) and Gross and Siklos (2020) to compute directional connectedness from an estimation of variance decomposition to construct a network. We consider a covariance stationary VAR (*p*) with an *N*-dimensional vector of sovereign CDS returns ${\varvec{r}}_{{\varvec{t}}} = \left( {r_{1t} , \ldots ,r_{nt} } \right)^{^{\prime}}$ as follows:

1 $$r_{t} = \mathop \sum \limits_{k = 1}^{p} \Phi_{k} r_{t - k} + \varepsilon_{t}$$

Here, $\varepsilon_{t} \sim iid\left( {0,\Sigma } \right)$ and $\Phi_{i}$ are the $N \times N$ parameter matrices with the lag length *p*.^3^ The moving average representation can be defined as follows:

2 $$r_{t} = \mathop \sum \limits_{k = 0}^{\infty } A_{k} \varepsilon_{t - k}$$

The $N \times N$ coefficient matrices $A_{k}$ are defined recursively as $A_{k} = \Phi_{1} A_{k - 1} + \Phi_{2} A_{k - 2} + \cdots + \Phi_{p} A_{k - p}$ to ensure the stability of coefficients. As the dimension increases, the identification of variance becomes difficult. Standard variance decompositions provided by Cholesky factorization rely on the order of the variables, considerably complicating measurements of directional connectedness (Billio et al. 2012). The generalized variance decomposition improved by Diebold and Yilmaz (2014) provides variable-order-invariant variance decompositions and accounts for correlated shocks to overcome these problems. We employ the generalized variance decomposition (Pesaran and Shin 1998) to measure directional network connectedness considering the high dimensionality of the sovereign CDS dataset. Therefore, network connectedness is an alternative measurement of the covariance risk of sovereign default. Thus, the effects of sovereign credit networks on currency risk premia should be investigated.

Following Diebold and Yilmaz (2014) and Yang (2019), we first estimate pairwise directional connectedness. It measures the contribution from sovereign CDS *j* to sovereign CDS *i* in terms of *H*-step-ahead generalized forecast error variance:

3 $$\theta_{ij}^{h} = \frac{{\sigma_{jj}^{ - 1} \mathop \sum \nolimits_{h = 0}^{H - 1} \left( {e_{i}^{^{\prime}} A_{h} \Sigma e_{j} } \right)^{2} }}{{\mathop \sum \nolimits_{h = 0}^{H - 1} \left( {e_{i}^{^{\prime}} A_{h} \Sigma A_{h}^{^{\prime}} e_{i} } \right)}},\quad H = 1,2, \ldots .$$

The covariance matrix ${\Sigma }$ is constructed by the error vector $\varepsilon$, the standard deviation of the error of the *j*th equation is denoted by $\sigma_{jj}$, and the selection vector $e_{i}$ has a value of 1 as the *i*th element and 0 if otherwise. Under the generalized variance decomposition framework, the variance shares do not add to 1. Accordingly, we can normalize Eq. (3) as follows:

4 $$C_{i \leftarrow j}^{H} = \frac{{\theta_{ij}^{g} \left( H \right)}}{{\mathop \sum \nolimits_{j = 1}^{N} \theta_{ij}^{g} \left( H \right)}}$$

where $C_{i \leftarrow j}^{H}$ denotes pairwise directional connectedness from sovereign CDS *j* to sovereign CDS *i*. Consequently, the total directional connectedness to sovereign CDS *i* of all other sovereign CDSs *j* is calculated as follows:

5 $$C_{i \leftarrow \bullet }^{H} = \frac{{\mathop \sum \nolimits_{j = 1,j \ne i}^{N} C_{i \leftarrow j}^{H} }}{{\mathop \sum \nolimits_{i,j = 1}^{N} C_{i \leftarrow j}^{H} }} = \frac{{\mathop \sum \nolimits_{j = 1,j \ne i}^{N} C_{i \leftarrow j}^{H} }}{N}$$

Similarly, the total directional connectedness from sovereign CDS *i* to all other sovereign CDSs *j* is calculated as follows:

6 $$C_{ \bullet \leftarrow i}^{H} = \frac{{\mathop \sum \nolimits_{j = 1,j \ne i}^{N} C_{j \leftarrow i}^{H} }}{{\mathop \sum \nolimits_{i,j = 1}^{N} C_{j \leftarrow i}^{H} }} = \frac{{\mathop \sum \nolimits_{j = 1,j \ne i}^{N} C_{j \leftarrow i}^{H} }}{N}$$

Finally, the total directional connectedness of the network is measured as follows:

7 $$C^{h} = \frac{{\mathop \sum \nolimits_{i,j = 1,j \ne i}^{N} C_{j \leftarrow i}^{H} }}{{\mathop \sum \nolimits_{i,j = 1}^{N} C_{j \leftarrow i}^{H} }} = \frac{{\mathop \sum \nolimits_{i,j = 1,j \ne i}^{N} C_{j \leftarrow i}^{H} }}{N}$$

For our analysis, we set a forecast horizon H = 10 with a lag width of 1. In addition, we employ elastic net shrinkage (Zou and Hastie 2005; Zou and Zhang 2009), an extended version of LASSO, to shrink, select, and estimate the VAR model to overcome the curse of dimensionality.

8 $$\hat{\beta }_{AEnet} = \mathop {\arg \min }\limits_{\beta } \left( {\mathop \sum \limits_{t = 1}^{T} \left( {r_{it} - \mathop \sum \limits_{k = 1}^{p} {\varvec{\beta}}_{{{\varvec{k}},{\varvec{i}}}}^{\user2{^{\prime}}} {\varvec{r}}_{{{\varvec{t}} - {\varvec{k}}}} } \right)^{2} + \lambda \mathop \sum \limits_{k = 1}^{p} \left[ {\left( {1 - \alpha } \right)\left| {{\varvec{\beta}}_{{{\varvec{k}},{\varvec{j}}}} } \right| + \alpha \left| {{\varvec{\beta}}_{{{\varvec{k}},{\varvec{j}}}} } \right|^{2} } \right]} \right)$$

Here, *i* = 1, …, *n*, and ***r*** are the sovereign CDS returns. As suggested by Zou and Hastie (2005), the elastic net penalty $\left( {1 - \alpha } \right)\left| {{\varvec{\beta}}_{{{\varvec{k}},{\varvec{j}}}} } \right| + \alpha \left| {{\varvec{\beta}}_{{{\varvec{k}},{\varvec{j}}}} } \right|^{2}$ becomes the LASSO penalty if $\alpha = 0$ and becomes a simple ridge regression if $\alpha = 1$. The tuning parameter $\lambda$ controls the overall magnitude of the penalty by controlling the number of penalized regressors. For example, when $\lambda = 0$, Eq. (8) becomes the standard ordinary least squares estimator. Then, we employ tenfold crossvalidation to determine $\alpha$ and $\lambda$ to obtain the lowest squared error. The optimal λ and α would be those that cause the least in-sample mean squared error in the model. Although this step is extremely time-consuming, it facilitates the estimation of the sovereign CDS network with quarterly samples without changing the dimensions.

We also visualize the global sovereign network based on the connectedness estimated from Eqs. (5) and (6) to obtain network centrality. Correspondingly, the estimated network consists of 41 nodes and 41 × 40 = 1640 links.^4^ The network elements are as follows: node name, color, size, and location and edge thickness. *Node name* is a three-digit abbreviation for each country. *Node color* represents geographical locations: Europe is red, South America is green, Asia is yellow, Africa is purple, North America is blue, and Oceania is orange. *Node size* is determined by the *to-other* connectedness measure and represents the influence of a node on other nodes or the network. *Node location* is determined using Fruchterman and Reingold’s (1991) force-directed algorithm. *Edge thickness* represents pairwise directional connectedness between two nodes. After developing the network graph, we consider four measurements of network centrality based on graph theory. Table 2 presents these measurements.

### Currency risk measure

We consider currency excess returns and forward spreads as proxies for currency risk premia to understand the effect of network centrality on currency risk premia. Following Akram et al. (2008), the currency excess return for a long position in country *i* by a US investor ($rx_{it + 1}$) can be expressed as follows:

9 $$rx_{it + 1} = f_{it} - s_{it} - \Delta s_{it + 1} = f_{it} - s_{it + 1}$$

where $f_{it}$ and $s_{it}$ are the log forward and spot exchange rate, respectively, in country *i* in terms of US dollars, $r_{it}$ is the 1-month log interest rate in country *i*, and $r_{t}$ is the 1-month log US interest rate. If the covered interest parity holds, then the forward spread $fs_{it}$ can be equal to $f_{it} - s_{it}$, which can be rewritten as $r_{it} - r_{t}$. Consequently, Eq. (9) can be rewritten as follows:

10 $$rx_{it + 1} = r_{it} - r_{t} - \Delta s_{it + 1}$$

This equation implies that a US investor who takes a long position on currency *i* at time *t* would receive the difference between the forward spread and the changes in the spot exchange rate at time *t* + 1. In particular, $\Delta s_{it + 1}$ is unknown at time *t* and is significantly predicted by the sovereign default network centrality.

### Empirical model

We regress the currency risk of a country ($ex_{i, t}$) at time *t* on the country fixed effects ($\alpha_{i}$), the lagged network centrality measures ($NC_{i, t - 1}$), the interaction terms of the binary variable (i.e., COVID-19 pandemic) with network centrality measures, and the lagged country-specific sovereign characteristics after obtaining all the necessary inputs. The country-specific sovereign characteristics are considered the control variables ($C_{i, t}$) at time *t*.

11 $$ex_{i, t} = \alpha_{i} + \beta_{1} NC_{i, t - 1} + \gamma C_{i, t} + u_{i, t}$$

where $ex_{i, t}$ denotes the forward spread $fs_{it}$ or currency excess returns $rx_{it}$. A larger value of $ex_{i, t}$ denotes a higher currency risk in a country for a long position. Therefore, if the coefficients are positive, then we expect that the aforementioned variables have a positive effect on currency risk. As suggested by Richmond (2019), a country with higher trade network centrality has lower currency risk premia and a lower interest rate. Bostanci and Yilmaz (2020) also provided evidence that the connectedness in the sovereign CDS market is highly correlated with the trade and capital flow. In this sense, the network centrality from the sovereign CDS market should provide similar results to the trade network centrality because the sovereign risk is generated from international trade (Chang et al. 2021). For example, the 3-month bond yield for one country is always considered a domestic risk-free rate. However, this yield is risky for foreign investors. Therefore, the developed global sovereign default network partially reflects the global trade network. Given the aforementioned facts, we expect a significant negative relationship between network centrality and currency risk for centralized countries. For example, if a country is an important or a centralized node in the network, then high network centrality allows the country to transmit its idiosyncratic sovereign risk into the network through its currency (Chen et al. 2020; Corte et al. 2021). Thus, the aforementioned negative relationship should be detected because a country with a higher network centrality has a stronger ability to transmit its risk into the foreign exchange system (Augustin et al. 2020).

Importantly, we do not consider time fixed effect in Eq. (11) because a part of the network centrality variations for each node is absorbed. Specifically, the average network centrality is stable although the network centrality for each node changes over time. The coefficients of network centrality only capture the currency risk relative to the average risk of all countries or average network centrality when time fixed effects are incorporated because the network centrality is stable. Then, the coefficients of network centrality provide evidence that network centrality has no effect on the change in currency risk. Mathematically, we developed a sovereign default network based on standardized connectedness, as mentioned in Eq. (4). The network centralities exhibit heterogeneity across the periods. However, the variations are partly eliminated under the time fixed effect because the network centrality measures the importance of the node in the network rather than that of the network itself. In this sense, the sovereign default network centrality is distinct from international trade network centrality as it is more cross-sectional characteristics. Therefore, we design the interaction term to capture the aforementioned variations in our regression with robust standard errors. Moreover, the influence of the country size on currency risk is not a concern because all the inputs in the baseline regression are standardized.

Table 3 presents the descriptive statistics obtained in this study. The mean currency excess return is − 0.0019, and its standard deviation is 0.0322. In comparison, the mean currency forward spread is 0.0025, and its standard deviation is 0.0119. These results indicate that the currency excess return is negative and has larger volatility than the currency forward spread. We consider the government debt ratio and total debt ratio measured by the external debt, which is the key factor affecting the exchange rate and sovereign risk, as the control variables. The average values of the sovereign characteristics are 0.2452 and 2.2636 for the government and total debt ratios, with the corresponding standard deviations of 0.1676 and 2.7672, respectively. The results indicate that the variations of the external debt are higher than those of the government debt ratio. Moreover, the average total debt ratio is more than 10 times higher than the average government debt ratio.

**Table 3:** Descriptive statistics

Variable	Mean	Median	Max	Min	Std.Dev
Dependent variable					
Currency excess return	− 0.0019	0.0000	2.946	− 0.3565	0.0322
Currency forward spread	0.0025	0.0011	0.4258	− 0.1591	0.0119
Network centrality					
In-strength	0.7285	0.7850	0.9999	0.000	0.1748
Out-strength	0.4230	0.4080	0.9966	0.000	0.2211
Closeness centrality	0.0051	0.0047	0.0152	0.0006	0.0025
Betweenness centrality	42.0883	78.5021	491.0000	0.000	74.3934
Eigenvector centrality	0.2876	0.0980	0.9999	0.000	0.3924
Sovereign characteristics					
Government debt ratio	0.2452	0.2048	0.7494	0.0009	0.1676
Total debt ratio	2.2636	1.6012	28.9474	0.1106	2.7672

Variable definitions are provided in Table 2

## Empirical results

### Sovereign default network

**Fig. 1:**
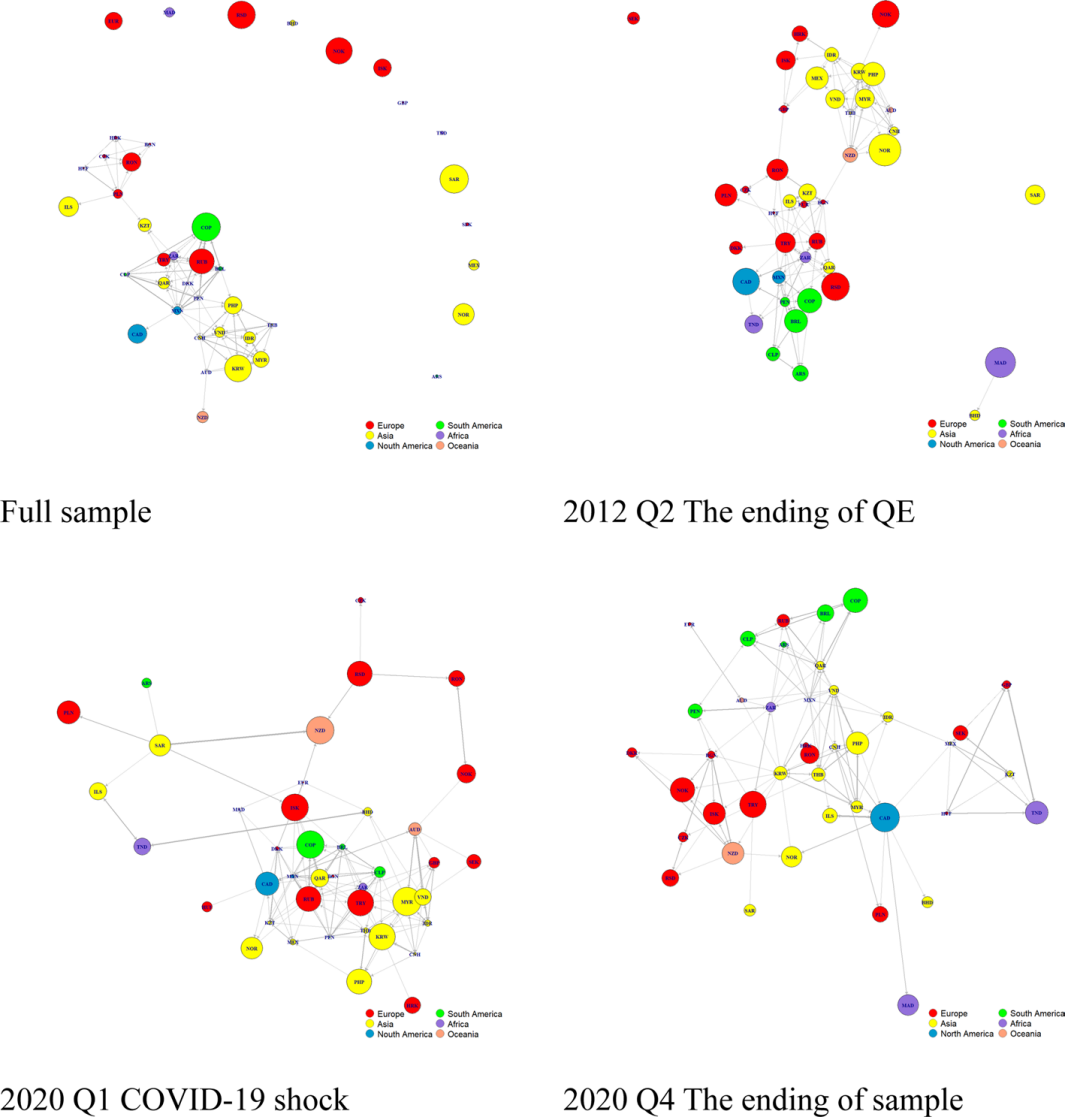
The plots of network for different market conditions

Figure 1 shows the sovereign default network developed in this study. For full-sample analysis, we identify a strong community-nested pattern. The nodes of sovereign CDS entities with similar economic conditions and geographic backgrounds tend to bunch together, particularly in Asia. For example, the Asian, European, and African groups are evidently distinguishable. Notably, the aforementioned network is developed according to a connectedness greater than the 95% quantiles. Accordingly, we do not report weaker connectedness although it exists. The economies take the central role in the network, indicating the systematic importance of the sovereign default network. The aforementioned economies are the senders or transmitters of sovereign risk to the global sovereign market.

Figure 1 displays three other network graphs for several key events to further illustrate the sovereign default network. For example, the second quarter of 2012 is the time point when the European Central Bank provides a second bailout package. Thus, this time point indicates the tipping point of the European debt crisis. We can witness the strengthening of the community-nested behavior of the sovereign CDS, and a clear geographical cutoff can be identified. Unsurprisingly, the COVID-19 pandemic is responsible for the massive exogenous shock to the sovereign default network in our sample. Therefore, we also illustrate the network graph for the first quarter of 2020. We depict the network graph for the fourth quarter of 2020, which marks the end of our sample, considering that the outcomes of the COVID-19 pandemic vary by country. Under the COVID-19 shock, the community-nested pattern disappears and is replaced with an interproximal pattern, which is evidence of the complexity of network structures. Thus, the COVID-19 pandemic makes the nodes in the network more centralized. By contrast, the last network graph appears to return to the community-nested pattern. Evidently, the network structures are significantly different. Thus, the communities exhibit different patterns for different periods, suggesting that the network centrality changes.

We observe the asymmetric effect of the degree centrality to consider the network centrality. This effect has a higher in-strength than out-strength.^5^ Other network centralities also provide evidence that the sovereign default network follows the core–periphery structure. Moreover, the standard deviation of network centrality is large, indicating that the network structures change significantly during the sample period. We observe in the three graphs that the network centrality is highly dependent on economic development and geographic location. However, the network centrality changes significantly during our sample period. Therefore, further investigation is required to understand the change in network centrality and its effect on currency risk premia.

**Fig. 2:**
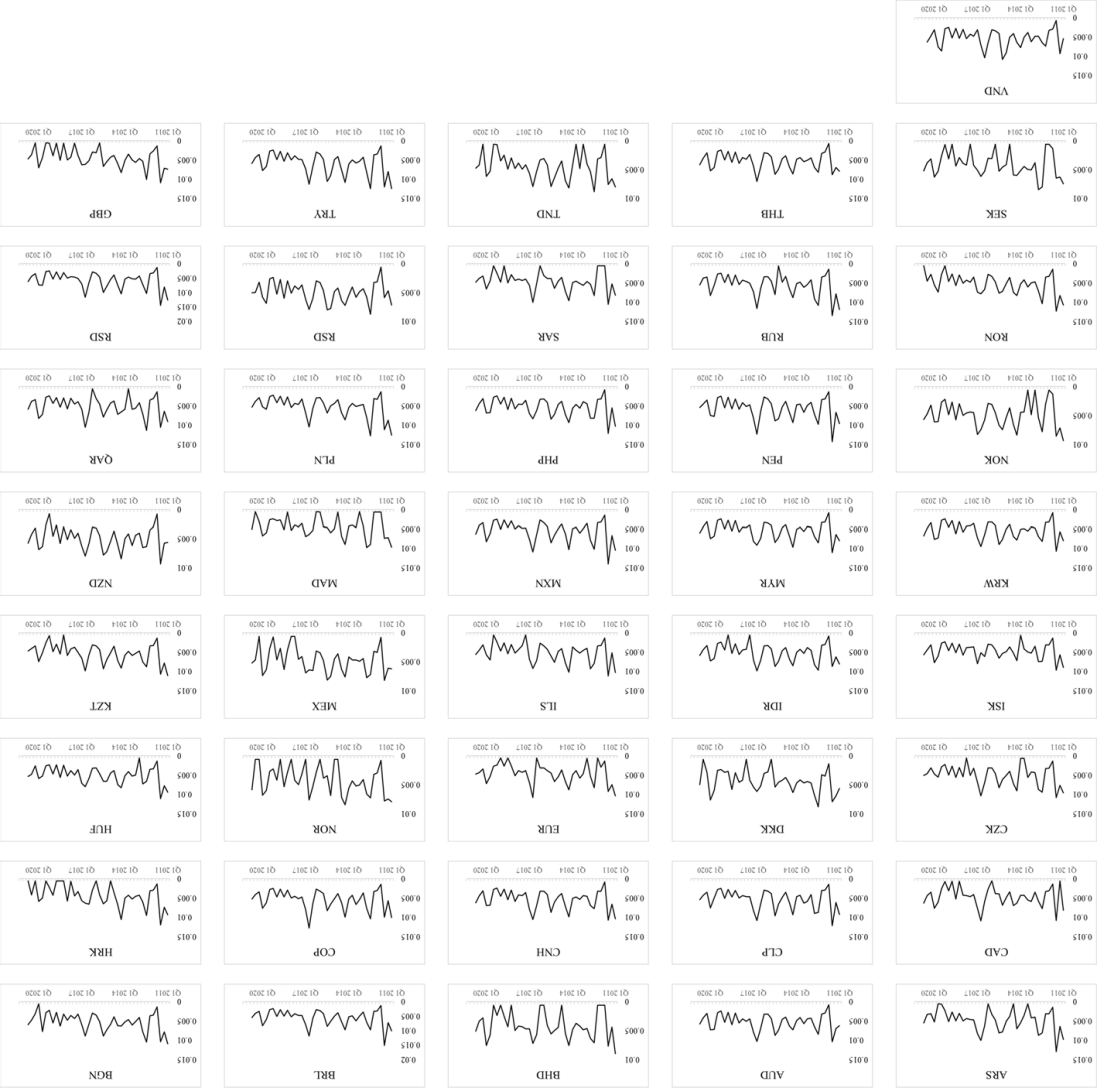
Time series of closeness centrality for each country

Figure 2 illustrates the closeness centrality of each country in the sample for a simple interpretation. Moreover, although the closeness centrality changes frequently, the peripheral countries are still highly consistent, which may explain the currency excess returns. Unlike trade networks, sovereign default networks are unstable. Sovereign CDS is an indicator of investors’ expectations regarding sovereign default events. Hence, changes in expectations regarding default expectations are reflected by the sovereign CDS price, which in turn causes sovereign risk spillover and changes sovereign default networks accordingly.

### Role of sovereign default network centrality on currency risk premia

We examine the effect of network centrality on currency risk premia (Table 4). We observe that the negative coefficients of closeness and betweenness centralities on the currency excess return are significant at the 5% level by allowing heterogeneous effects across countries. However, we do not find any significant evidence of an association between network centrality and currency forward spread. Thus, our findings are consistent with those of Yamani (2019). In particular, the role of closeness centrality is crucial because the currency excess return is reduced by 0.375 when closeness centrality increases by one standard deviation. Closeness centrality plays a key role in the absorption of idiosyncratic sovereign risk into the sovereign default network. From the Twin Ds perspective, core countries experience less devaluation of their currencies when other countries experience sovereign default (Augustin et al. 2020; Chernov et al. 2021). The sovereign default network’s influence on countries’ currency excess returns is more significant compared with the cross-sectional standard deviation of countries’ currency excess returns, which is 0.032. Sovereign default network centrality has a higher influence on the currency excess return than trade network centrality (Richmond 2019).

**Table 4:** Network centrality and currency sovereign risk

	(1)	(2)	(3)	(4)	(5)
Dependent variable A: currency excess return					
Constant × 10^−3^	− 3.697 (3.202)	− 1.561 (1.431)	− 0.033 (0.564)	− 1.256*** (0.212)	− 1.195** (0.057)
Centrality × 10^−3^	2.544 (4.441)	0.764 (3.314)	− 375.130*** (110.619)	− 0.015** (0.005)	2.392 (1.997)
Country FE	Yes	Yes	Yes	Yes	Yes
No. of obs	4797	4797	4797	4797	4797
Adj. R^2^ within	0.001	0.000	0.001	0.001	0.001
Dependent variable B: currency forward spread					
Constant × 10^−3^	5.223** (2.272)	4.425*** (1.125)	2.538*** (0.368)	2.717*** (0.145)	2.747*** (0.280)
Centrality × 10^−3^	2.531 (4.334)	4.190 (2.677)	11.211 (72.096)	0.006 (0.04)	0.926 (0.974)
Country FE	Yes	Yes	Yes	Yes	Yes
No. of obs	4797	4797	4797	4797	4797
Adj. R^2^ within	0.001	0.001	0.001	0.001	0.001

From column 1–5 the network centrality denote in-strength, out-strength, closeness, betweenness, and eigenvector centrality, respectively. The Huber–White heteroskedasticity-robust standard errors adjusted for country clustering are provided in parentheses. *** and ** indicate significance at the 1% and 5% levels

From the network perspective, a higher network centrality indicates higher resilience to the propagation of idiosyncratic sovereign risk into the sovereign default network. As currency risk premia are a part of the idiosyncratic sovereign risk, the corresponding currency risk is reduced because of the higher resilience of the network. Thus, a densely connected network is more likely to absorb idiosyncratic risk and transform it into systematic risk (Acemoglu et al. 2015; Caporin et al. 2018; Chen et al. 2020). However, we did not find evidence that eigenvector centrality has a significant influence on the currency excess return. Importantly, we do not discuss further the magnitude of their coefficients given the relative measurements on the network centrality.

**Table 5:** Network centrality and currency sovereign risk under the COVID-19 pandemic

	(1)	(2)	(3)	(4)	(5)
Dependent variable A: currency excess return					
Constant × 10^−3^	− 3.426 (2.797)	− 1.561 (1.431)	− 0.349 (0.523)	− 1.065*** (0.204)	− 0.601 (0.515)
Centrality × 10^−3^	− 1.411 (3.911)	− 2.091 (2.954)	− 422.029*** (105.253)	− 0.017*** (0.004)	− 4.044* (2.076)
Centrality × COVID19	0.016*** (0.002)	0.020*** (0.004)	2.516*** (0.311)	0.054** (0.027)	0.004* (0.0021)
Country FE	Yes	Yes	Yes	Yes	Yes
No. of obs	4797	4797	4797	4797	4797
Adj. R^2^ within	0.009	0.000	0.013	0.002	0.001
Dependent variable B: currency forward spread					
Constant × 10^−3^	5.032** (2.026)	4.425*** (0.998)	2.622*** (0.308)	2.756*** (0.139)	2.806*** (0.235)
Centrality × 10^−3^	− 1.151 (4.414)	− 3.796 (2.513)	− 9.605 (6.851)	− 0.003 (0.002)	− 1.189 (0.094)
Centrality × COVID19	− 0.003 (0.003)	− 0.001 (0.001)	− 0.181 (0.207)	− *0.036 (0.025)*	− 0.001 (0.207)
Country FE	Yes	Yes	Yes	Yes	Yes
No. of obs	4797	4797	4797	4797	4797
Adj. R^2^ within	0.003	0.005	0.001	0.007	0.002

From column 1–5 the network centrality denote in-strength, out-strength, closeness, betweenness, and eigenvector centrality, respectively. The numbers in italic denotes its value should time 10^−3^. The Huber-White heteroskedasticity-robust standard errors adjusted for country clustering are provided in parentheses. ***, **, * indicate significance at the 1%, 5%, 10% levels

Next, we consider the COVID-19 pandemic as an exogenous shock to examine its effect on currency risk premia. Hence, we introduce an interaction term between network centrality and the COVID-19 shock into Eq. (11). Table 5 presents the corresponding empirical results obtained using Eq. (11). Specifically, we define the COVID-19 pandemic period as the period from the first quarter of 2020 to the end of the fourth quarter of 2020. The coefficients of the interaction term between the COVID-19 pandemic indicator and network centrality are significantly positive. This case suggests that the influence of network centrality during the COVID-19 pandemic increases the currency excess return. However, we are unable to find significant empirical evidence of an association between network centrality and forward spread. Similar to the results of Corte et al. (2021), our results also indicate that exposure to global sovereign risk shocks contributes to currency excess returns rather than forward spread. Thus, sovereign risk is independent of interest differentials and the carry factor.^6^

In particular, the interaction term of degree centrality and COVID-19 is significantly positive. This case implies that financial contagion occurs from the sovereign CDS market to the foreign exchange market. This result may be explained by an increase in idiosyncratic risk because of the changes in network centrality in the global sovereign CDS market during the COVID-19 pandemic. Notably, the absolute value of coefficients and their significance on the network centrality–related variable increase after adding the COVID-19 pandemic indicator. This case suggests that the COVID-19 pandemic shock is a special episode in the sample. During the COVID-19 pandemic, an increase in network centrality indicated an increase in the resilience of core countries to sovereign risk shocks and idiosyncratic contagion (Chen et al. 2020). The findings also support those of Augustin et al. (2020), who observed less devaluation of core countries’ currencies owing to the shock from the COVID-19 pandemic.

**Table 6:** Network centrality and currency excess return under the different foreign exchange regimes

	(1)	(2)	(3)	(4)	(5)
Dependent variable: currency excess return (floating)					
Constant × 10^−3^	− 4.589 (4.197)	− 1.851 (1.779)	− 0.949 (0.650)	− 1.332*** (0.262)	− 0.721*** (0.762)
Centrality × 10^−3^	2.651 (5.734)	− 1.960 (4.192)	− 395.036*** (126.563)	− 0.019*** (0.005)	− 4.968* (2.631)
Centrality × COVID19	0.016*** (0.002)	0.021*** (0.004)	2.647*** (0.362)	0.036*** (*0.003*)	0.005 (0.005)
Country FE	Yes	Yes	Yes	Yes	Yes
No. of obs	3627	3627	3627	3627	3627
Adj. R^2^ within	0.009	0.006	0.012	0.002	0.002
Dependent variable: currency excess return (non-floating)					
Constant × 10^−3^	− 0.088 (0.098)	− 0.001 (0.499)	− 1.497* (0.751)	− 0.188*** (0.187)	− 0.292 (0.191)
Centrality × 10^−3^	− 1.981 (1.371)	− 1.924 (1.215)	− 507.558** (181.453)	− 0.012** (0.004)	− 0.026 (0.99)
Centrality × COVID19	0.013*** (0.004)	0.015** (0.005)	2.051*** (0.591)	0.095*** (0.022)	0.024 (0.097)
Country FE	Yes	Yes	Yes	Yes	Yes
No. of obs	1170	1170	1170	1170	1170
Adj. R^2^ within	0.014	0.007	0.020	0.011	0.000

From column 1–5 the network centrality denote in-strength, out-strength, closeness, betweenness, and eigenvector centrality, respectively. The numbers in italic denotes its value should time 10^−3^. The Huber-White heteroskedasticity-robust standard errors adjusted for country clustering are provided in parentheses. ***, **, * indicate significance at the 1%, 5%, 10% levels

We examine the role of a country’s heterogeneity on its currency excess return to increase the robustness of our empirical results.^7^ Figure 2 shows the core–periphery structure. Therefore, we divide our sample into two groups according to whether the relevant currency is floating. Specifically, foreign exchange regimes are classified according to the 2019 IMF Annual Report on Exchange Arrangements and Exchange Restrictions, with the results provided in Table 1. Overall, our sample includes 31 floating currencies and 10 nonfloating currencies. The distinction between floating and nonfloating currencies serves three purposes. First, a country with a nonfloating currency may have strict international capital regulations that make its sovereign CDS market relatively isolated from the global sovereign default network. Second, a trading strategy (i.e., carry trade) may be unavailable in a country with a nonfloating currency although the forward currency market exists. Third, countries in the nonfloating currency group are usually not industrialized countries and exhibit a high sovereign risk. Table 6 shows the empirical findings. The previous findings still hold although the degree of coefficients may differ. For the nonfloating currency group, we detect a stronger influence on currency excess return from closeness centrality than from betweenness centrality. These findings indicate that the countries in the nonfloating currency group are peripherals. We use the control variables in Eq. (11) to make our findings more robust. Table 7 shows the obtained empirical results, and the results are still valid.

**Table 7:** Network centrality and currency excess return with the controls

	(1)	(2)	(3)	(4)	(5)
Dependent variable: currency excess return					
Constant × 10^−3^	− 2.002 (1.463)	− 0.967 (2.132)	− 0.529 (1.786)	− 1.159 (1.440)	− 0.998 (1.554)
Centrality × 10^−3^	1.911 (4.338)	− 2.155 (3.326)	− 418.517*** (111.862)	− 0.018*** (0.004)	− 4.204* (0.231)
Centrality × COVID19	0.017*** (0.002)	0.022*** (0.004)	2.781*** (0.322)	0.054* (*0.029*)	0.006 (0.005)
Debt ratio × 10^−3^	− 1.415 (6.271)	− 2.035 (6.381)	0.774 (5. 901)	0.092 (5.681)	1.058 (5.686)
Total debt ratio × 10^−5^	− 0.842 (0.742)	− 0.733 (0.647)	− 0.044 (0.043)	− 0.068 (0.040)	0.522 (0.421)
Country FE	Yes	Yes	Yes	Yes	Yes
No. of obs	4329	4329	4329	4329	4329
Adj. R^2^ within	0.010	0.007	0.014	0.002	0.002

From column 1–5 the network centrality denote in-strength, out-strength, closeness, betweenness, and eigenvector centrality, respectively. The numbers in italic denotes its value should time 10^−3^. The Huber-White heteroskedasticity-robust standard errors adjusted for country clustering are provided in parentheses. *** and * indicate significance at the 1% and 10% levels

### Asset pricing implications

The analysis described thus far provides direct evidence that sovereign default network centrality can be a critical pricing factor in determining the currency excess return but not the forward spread. We follow the methods of Lustig and Verdelhan (2007) and Lustig (2011) in constructing a portfolio sorted by the closeness centrality of the sovereign default network to take advantage of this pricing factor.^8^ This step allows us to identify the common component of currency risk related to the sovereign default network. As forward spread is irrelevant to the sovereign default network, the pricing factor of network centrality is independent of the unconditional carry trade risk factor. These findings support those of Corte et al. (2021). Hence, we do not consider the carry trade strategy to be a benchmark. Instead, we consider the currency momentum strategy as a benchmark given that the previous currency excess return is also affected by the sovereign default network. Furthermore, we follow the study of Richmond (2019) who also employed the Sharpe ratio to evaluate strategies. Consequently, we construct five portfolios by using two sorting indicators: prior quarter closeness centrality and previous currency excess return. Provided that these two indicators are observable at time *t*, exploiting the arbitrage opportunity and rebalancing them monthly are in our best interest.^9^

**Table 8:** Portfolios sorted on network centrality

	Portfolio 1	Portfolio 2	Portfolio 3	Portfolio 4	Portfolio 5	DOL
Panel A: closeness centrality: $v_{it - 1}$ (f = 1, h = 1) full sample						
Mean	− 0.906 [− 0.85]	− 0.540 [0.60]	− 0.350 [− 0.43]	− 1.211 [− 1.46]	1.203 [1.94]	2.109 [2.15]
Sharpe ratio						0.172
Panel B: closeness centrality: $v_{it - 1}$ (f = 1, h = 1) floating sample						
Mean	− 1.091 [− 0.91]	− 0.786 [− 0.71]	− 0.939 [− 0.86]	− 0.595 [− 0.59]	1.446 [2.09]	2.437 [2.15]
Sharpe ratio						0.228
Panel C: currency momentum: $rx_{it - 1}$ (f = 1, h = 1) full sample						
Mean	− 1.539 [− 1.31]	− 0.555 [− 0.62]	− 0.692 [− 0.92]	− 0.348 [0.43]	0.375 [0.40]	1.914 [1.36]
Sharpe ratio						0.126
Panel D: currency momentum: $rx_{it - 1}$ (f = 1, h = 1) floating sample						
Mean	− 0.870 [− 0.65]	− 0.555 [− 0.56]	− 0.018 [− 0.02]	− 0.074 [− 0.08]	0.430 [0.43]	1.300 [0.91]
Sharpe ratio						0.084

The portfolios are constructed by previous closeness centrality (*v*) or previous currency excess returns (log risk premia, *rx*). We set portfolio formation period and holding period equal to 1 month. The last column in Panel A and B is the difference between the portfolio 5(peripheral) and the portfolio 1(core) for NCF strategy. The last column in Panel C and D is the difference between the portfolio 5(winner) and the portfolio 1(loser) for MOM strategy. The value in square brackets is *t*-statistics

Table 8 reports the results of sorting the portfolios under the 1-month formation period and 1-month holding period.^10^ Panel A presents portfolios sorted by closeness centrality for the full sample. Although returns from the portfolio of core countries to the portfolio of peripheral countries are not monotonically increasing, the long–short trading strategy generates a return of 211 basis points with an annualized Sharpe ratio of 0.172.^11^ In comparison, for the full-sample analysis, the currency momentum strategy generates an annualized return of 191 basis points with an annualized Sharpe ratio of 0.126. Thus, our proposed strategy outperforms the currency momentum strategy. In particular, portfolio 4 generates the lowest return across the portfolios. This abnormality disappears when we implement the floating currency sample presented in panel B. The annualized return of the long–short trading strategy is 244 basis points with an annualized Sharpe ratio of 0.228 for this sample. For the floating currency sample, the currency momentum strategy generates an annualized return of 130 basis points with an annualized Sharpe ratio of 0.084. Overall, our proposed strategy outperforms the currency momentum strategy.^12^

We develop long–short risk factors (Lustig et al. 2011) for each sorting variable by employing the floating currency sample to strengthen our findings. We then define the long–short trading strategy according to network centrality as the NCF and the momentum strategy as the MOM. Importantly, the NCF and MOM can be interpreted as idiosyncratic risk factors in the sense that currencies’ excess return is generated by idiosyncratic risk from the sovereign default network. Table 9 presents the annualized summaries of the aforementioned risk factors for the full and floating samples. Panel A presents the relevant descriptive statistics. For the full sample, the NCF and MOM provide similar average returns, whereas the NCF shows higher Skewness and Kurtosis. For the floating currency sample, the average returns, Skewness, and Kurtosis of the NCF are considerably higher than those of the MOM. These results are consistent with those presented in Table 8. Panel B provides the correlation matrix between them to understand the relationship between the NCF and MOM. A significant positive relationship is evident between the aforementioned factors in the floating currency sample. However, the weak correlation between NCF and MOM also indicates that closeness centrality may be a new pricing factor for currency excess returns.

**Table 9:** Summary statistics of currency risk factors

	NCF full	NCF floating	MOM full	MOM floating
Panel A: descriptive statistics				
Mean	0.21	0.22	0.25	0.13
Std. Dev	1.06	0.80	1.11	1.54
Skew	0.63	1.34	0.44	0.32
Kurtosis	6.92	9.69	4.16	4.71
Observations	116	116	116	116
Panel B: correlation matrix				
NCF full	1			
NCF floating	0.905***	1		
MOM full	0.009	0.171*	1	
MOM floating	0.223**	0.251***	0.430***	1

***, **, * indicate significance at the 1%, 5%, 10% levels. *NCF* denotes the degree of closeness centrality

We run a simple time-series regression to further explore the effect of the NCF on the currency momentum trade as follows:

12 $$rx_{i, t}^{P} = \alpha_{i} + \beta_{i} NCF_{ t} \left( {MOM_{ t} } \right) + \epsilon_{i, t}$$

where $rx_{i, t}^{P}$ denotes the excess return from portfolios 1–5 (*i* = 1, 2, 3, 4, 5) determined using the NCF or MOM. As the closeness centrality enables the direct measurement of the node’s influence on the entire sovereign default network, a highly negative currency excess return indicates a low-risk loading from the currency, which is always associated with the high resilience of idiosyncratic contagion of sovereign risk for the currency. Thus, the currencies in the core group are usually risk absorbers in the network. To test this finding, we use Eq. (12) for the floating currency sample. Table 10 shows the relevant empirical results.

**Table 10:** Time series regressions of portfolios on NCF under the floating currency sample

	Portfolio 1	Portfolio 2	Portfolio 3	Portfolio 4	Portfolio 5
*Panel A: closeness centrality*					
$\alpha \times 10^{ - 3}$	1.123* (0.590)	0.426 (0.778)	0.587 (0.749)	0.735 (0.748)	1.123* (0.590)
$\beta$	− 0.035 (0.062)	0.437*** (0.082)	0.650*** (0.079)	0.659*** (0.079)	0.964*** (0.062)
Adj.R^2^	0.015	0.192	0.367	0.374	0.675
Observations	116	116	116	116	116
*Panel B: currency momentum*					
$\alpha \times 10^{ - 3}$	0.841 (0.954)	0.628 (0.729)	0.974 (0.654)	0.742 (0.698)	1.191 (0.785)
$\beta$	0.743*** (0.101)	0.517*** (0.077)	0.468*** (0.069)	0.381*** (0.073)	0.394*** (0.082)
Adj.R^2^	0.317	0.227	0.281	0.183	0.158
Observations	116	116	116	116	116

The regression specification is $rx_{i, t}^{P} = \alpha_{i} + \beta_{i} NCF_{ t} + \epsilon_{i, t}$. *NCF* denotes the degree of closeness centrality. Standard error is in parentheses. ***, * indicate significance at the 1% and 10% levels

Panel A presents the regression results for the portfolio sorted by the closeness centrality. The coefficients of unexplained excess returns are statistically positive. Moreover, the R-squared values are high, with their value monotonically increasing from the core portfolio 1 to the peripheral portfolio 5. However, the risk loading factor, namely, the NCF, is not the pricing factor for the core portfolio, portfolio 1. Thus, the peripheral countries are not well integrated into the global sovereign default network, causing their currencies to be risk generators. Panel B presents the results of the portfolio sorted by the previous currency excess return, as provided by the currency momentum strategy. The results presented in panel B are similar to those presented in panel A, indicating that the NCF can significantly explain the MOM.^13^ The results indicate that currency excess returns are driven by countries’ exposure to global sovereign networks and that idiosyncratic sovereign risk is less relevant. The extent to which currencies are resilient to idiosyncratic contagion is dependent on countries’ centrality in the global sovereign network. These results suggest that NCF captures some information as momentum. Moreover, these benchmark strategies, which are widely used by investors, can be improved by using information regarding sovereign default networks. Thus, cross-sectional variation in default expectations regarding sovereign CDS may partially explain currency momentum.

## Conclusion

We construct a sovereign default network through the connectedness measurement by employing a high-dimensional VAR model with a LASSO estimator. We identify that the sovereign default network is the core–peripheral structure for the full-sample analysis. We also examine the shock induced by the COVID-19 pandemic on the sovereign default network. This pandemic makes the network structure more condensed. The study findings motivate us to uncover its relationship with currency risk premia.

Therefore, we develop four network centrality measures, namely, degree, closeness, betweenness, and eigenvector centralities, to investigate the determinants of the forward spread and currency excess return. The findings suggest that closeness and betweenness centralities are significant determinants of currency excess return but not of carry trade return. We also develop a trading strategy by using the previous closeness centrality to take advantage of these two pricing factors. This strategy generates a higher return and Sharpe ratio than the currency momentum strategy.

Our findings shed light on the alternative sources of risk exposure to the currency excess returns. Unlike the international trade network, the sovereign default network shows low persistence owing to the heterogeneous default expectations. Specifically, the value of one currency depends not only on the quality of the fundamental of its credit but also directly on the role it played in the sovereign credit risk spillover mechanism. Accessing the network centrality pricing factor helps us to understand how sovereign risk acts across markets and affects currency momentum. We present an alternative explanation for the currency momentum strategy based on a network perspective.

More broadly, our analysis helps researchers and investors recognize how the network structure of assets can predict future returns. From an academic perspective, network centrality provides an alternative method for exploring the economic importance of the Twin Ds. Between-country differences in sovereign credit risk are related to the structures of sovereign default networks, indicating a connection between the cross-sectional variations in default expectations and the variation in spillover. From an investor perspective, closeness centrality serves as a pricing factor that differs from the carry factor and should be utilized in trading strategies and the risk management process. When constructing global currency portfolios, the structures of sovereign default networks should be considered. Based on the empirical findings of this study, macrofinance theory should be further developed to link sovereign default networks and currency excess returns.

## Data Availability

Not applicable.

## Abbreviations

AIC: Akaike information criterion
CDS: Sovereign credit swap
LASSO: Least absolute shrinkage and selection operator
MOM: Momentum risk factor
NCF: Network centrality factor
Twin Ds: Default and devaluation
UIP: Uncovered interest rate parity
VAR: Vector-autoregression

## Footnotes

1 The sample of the US-dollar-based sovereign CDS comprises data with a long span so that broad coverage can be achieved. Moreover, if no US-dollar-based sovereign CDS is available, we transform the local-currency-based sovereign CDS into a US-dollar-based sovereign CDS according to the spot exchange rate. Therefore, we do not further consider the local-currency-based sovereign CDS.

2 Usually, the VAR model requires the number of time series to be larger than the number of cross-sectional series. Quarterly frequency is suitable for our analysis.

3 We apply the Akaike information criterion (AIC) to select the lag length, which is two.

4 We only present the network with node connectedness values greater than the value of the 95% quantile for simple interpretations. The full sample analysis revealed that the 95% quantile of node connectedness was 3.302%.

5 According to the connectedness measure, the in- and out-strength should be the same. However, when we set the 95% percentile as 4.617% for each link and delete the insignificant links, we obtain the results presented in Table 3.

6 Corte et al. (2021) were also open to the question of driving reasons between sovereign credit risk and currency markets for further discussions. Corte et al. (2021) demonstrated that sovereign risk factors are priced in the cross-section of currency returns and not subsumed by the carry factor.

7 Hereafter, we do not further consider the case of forward spread, given the empirical evidence provided in Tables 4 and 5, which indicates the NCF is independent of the carry trade.

8 Betweenness centrality provides results that are similar to but have a lower significance than those of closeness centrality. These results are available upon request.

9 We admit the trading strategies may appear to be unprofitable when considering transaction costs in the practice.

10 Limiting the formation and holding periods to 6 months provides similar results with increasing significance. The results are available upon request.

11 The low interest rate after the 2008 global financial crisis may account for the small return and Sharpe ratio.

12 Although the long–short position of our proposed strategy is the opposite of that of the currency momentum strategy, the comparison is still meaningful because sorting factors can be considered two sides of the same coin.

13 The COVID-19 pandemic does not change the sign of the NCF because our portfolios are rebalanced monthly. The results are available upon request.
